# Integrating AI-based triage in primary care: a qualitative study of Swedish healthcare professionals’ experiences applying normalization process theory

**DOI:** 10.1186/s12875-025-03057-9

**Published:** 2025-11-04

**Authors:** Ingrid Larsson, Elin Siira, Jens M Nygren, Lena Petersson, Petra Svedberg, Per Nilsen, Margit Neher

**Affiliations:** 1 School of Health and Welfare, Halmstad University, Box 823, Halmstad, SE- 301 18 Sweden; 2 Department of Health, Medicine and Caring Sciences, Division of Public Health, Faculty of Health Sciences, Linköping University, Linköping, Sweden

**Keywords:** Artificial intelligence, Primary care, Healthcare professionals, Implementation science, Normalization process theory, AI-based triage application, Qualitative research

## Abstract

**Background:**

Given the growing challenges in primary care, including high demand and workforce shortages, artificial intelligence (AI)-based triage applications are being explored as a means of alleviating workloads. While the potential of AI in this context is widely acknowledged, there is still limited empirical research on how such tools become embedded in routine practice, especially from healthcare professionals’ perspectives. This study focused on exploring healthcare professionals’ experiences of using an AI-based triage application in primary care.

**Methods:**

The study had a qualitative design with a deductive approach, involving 14 healthcare professionals (physicians, nurses, psychologists, and a social worker). Data were collected through semi-structured interviews. The data were analyzed through directed qualitative content analysis and categorized in accordance with normalization process theory (NPT).

**Results:**

The results of this study were framed by the NPT constructs: Coherence, Cognitive Participation, Collective Action, and Reflexive Monitoring. Professionals aimed to achieve *Coherence* by making sense of the AI triage application’s purpose and potential role in practice; however, insufficient initial information was reported to hinder a full understanding and meaningful engagement with the tool. The work of building and sustaining engagement (*Cognitive Participation*) was challenged by staff’s perceptions that use of the triage application was optional: this hindered the development of a “community of practice”.

During *Collective Action*, professionals tended to rely more on patients’ free-text descriptions than on the AI-generated summaries, reflecting concerns about the application’s adequacy, compared to clinical judgment. Finally, *Reflexive Monitoring* revealed persistent uncertainty about the application’s value, with professionals questioning its usefulness, effectiveness, and equitable accessibility across patient groups.

**Conclusions:**

This study found that, although the AI-based triage application appeared, at first, to be integrated into primary care practice, it was not embedded fully within professional and organizational routines. Despite a broad acceptance of digitalization among healthcare professionals, several barriers to meaningful use were identified. These included concerns about insufficient organizational and policy support, which hindered the application’s full integration into everyday workflows. The study results suggest that further efforts are needed to overcome these barriers and support the successful normalization of the AI-based triage application into routine practice.

**Supplementary Information:**

The online version contains supplementary material available at 10.1186/s12875-025-03057-9.

## Background

Primary care is a cornerstone of effective healthcare systems, serving as the first point of contact for patients and playing a crucial role in care coordination [1]. In Sweden, equitable access to healthcare is not only a fundamental principle but also a legal obligation, as stipulated in the Swedish Healthcare Act [2]. Triage is a key function of primary care coordination that includes the collection of patients’ medical histories, traditionally conducted via telephone or in person, to determine the most appropriate next steps in their care journey [3, 4].

Artificial intelligence (AI)-based triage applications have been proposed as a means of alleviating the workload of healthcare professionals, by automating key steps in the triage process. These steps include collecting patient medical histories and directing patients to appropriate treatment pathways. Such automation has the potential to enhance both the safety and efficiency of healthcare delivery [5–9]. By interpreting clinical data, AI can streamline administrative tasks and improve operational efficiency [10, 11]. Additionally, AI can support healthcare professionals in their core responsibilities while contributing to the generation of new knowledge in clinical practice [12, 13].

Research highlights that digital applications for patient management enhance patients’ access to primary care and enable care that is more on the patient’s terms [14–18]. However, these studies also reveal ambivalence among primary care professionals, who demonstrate both acceptance of using digital applications and concerns, including apprehension and fear [17]. This hesitancy may contribute to the slower-than-expected adoption of AI in primary care [19].

Despite the considerable promise of AI systems demonstrated in studies, a comprehensive literature review [20] shows that research on AI systems that automate medical history-taking and triage in clinical settings is still in its early stages. This underscores a significant gap between the development of AI models and their practical application, aligning with the conclusions of previous research on AI in healthcare [21, 22]. Most existing research focuses on prototyping, development, or validation, with relatively few studies examining the efficacy of AI systems in real-world clinical settings [23]. Cummings (2021) argues that failures of AI in safety-critical settings can generally be attributed to a fundamental inability of AI technology to take contextual factors into account, along with a lack of general understanding regarding the necessary criteria to determine the maturity of AI-embedded systems [24].

Despite growing interest in AI applications for triage and medical history-taking, empirical studies based in real-world primary care settings are scarce, particularly those investigating healthcare professionals’ perspectives [8]. While some research evaluates their effectiveness [9, 25, 26] 10.1136/bmjhci-2019-100114, explores system features [27], or investigates nurses’ experiences of digital triage in primary healthcare [28] these studies are often removed from the day-to-day realities of clinical practice. Generating practice-informed knowledge can support implementation efforts, enhance the integration of these technologies in clinical workflows, help realize the full potential of AI to improve healthcare safety and efficiency, and alleviate the administrative burden on healthcare. Critical analyses of empirical data are needed not only to inform implementation, but also to be able to anticipate new challenges, such as increased cognitive and administrative burden, lack of transparency in AI decision-making, algorithmic bias, or potential disruptions to the clinician–patient relationship [29, 30].

To address the identified research gap, this study explores primary care professionals’ experiences of the implementation and practical use of an AI-based triage application. Understanding how such applications are integrated into real-world primary care is essential to identify the factors that influence their sustained use [31]. Successful implementation requires not only technical integration but also alignment with local, organizational and cultural contexts [32], as well as awareness of the social processes that drive their normalization [33]. According to normalization process theory (NPT), these processes include continuous sense-making, collective engagement, the operationalization of new practices and reflective appraisal of their impact [34]. Guided by the NPT as a theoretical framework, this study aimed to explore the experiences of healthcare professionals, concerning how the AI-based triage application became embedded in practice.

## Methods

### Design

This study, whose aim was to explore the experiences of healthcare professionals concerning the integration and embedment in practice of an AI-based triage application, had a qualitative design that employed directed qualitative content analysis [35]. This deductive approach involved analyzing data through a theoretical lens and using constructs from the NPT [34]. The study is reported in accordance with the Consolidated Criteria for Reporting Qualitative Research (COREQ) 32-item checklist [36] to ensure trustworthiness (Additional File 1).

NPT provides a framework to understand the dynamics of implementing, embedding, and integrating new technology or complex interventions in clinical practice [34, 37]. The theory was conceived in response to limitations in existing implementation science theories, which often focused narrowly on individual behavior change or broad contextual determinants, while overlooking the everyday work through which new practices are integrated into routine settings. NPT was initially designed to examine the implementation of complex healthcare interventions, such as telehealth services, clinical guidelines and digital health tools, and to understand the social processes that support or hinder their uptake. Grounded in empirical research and informed by grounded theory methods, NPT draws theoretical insights from sociology. The theory was formalized through a series of studies, including qualitative research on the implementation of telemedicine and evidence-based guidelines [34].

NPT is centered on understanding social phenomena defined by four theoretical constructs which characterize mechanisms that are energized by investments made by individuals and organizations. The theory was deemed to be appropriate for the current study because it describes implementation at stages beyond early implementation, to the point where an intervention becomes routinely embedded in the work practices of individuals and organizations (i.e., is normalized) [38]. The leaders in the study context had procured and introduced the AI-based application into the system and/or organization(s) at least 3–5 years before the data collection, and implementation was deemed to be in the sustainment phase [33, 39].

NPT theory consists of four constructs that represent the social actions people engage in during the implementation of a new practice: individual and collective sense-making (*Coherence*), building and sustaining a new community of practice (*Cognitive participation*), enacting the practice (*Collective Action*) and appraising its effects (*Reflexive monitoring*) [34].

### Setting and technology

In Sweden, publicly funded healthcare is administered at the national, regional, and municipal levels, and the responsibility for primary care lies primarily with the country’s 21 regions. Digital technology is seen as a solution to addressing healthcare challenges, as outlined in a policy agreement between the Swedish government and the Swedish Association of Local Authorities and Regions on healthcare digitalization. Over the past decade, digital care solutions have been implemented, particularly in primary care, to assist with tasks like medical history-taking, referrals, digital examinations, diagnosis, and treatment [40, 41]. Nurses usually perform the initial triage and health guidance. After reading the triage forms and messages pre-filled by patients, they assess the patient’s need for health guidance and follow up by either synchronous (telephone-) or asynchronous text-based communication via SMS. AI technology has been proposed for use in areas such as medical history-taking, diagnosis, and decision support [42], but actual application of the technology regarding triage has remained limited in clinical primary care settings.

The AI-based triage application in this study was developed by a commercial company, with whom the authors did not have any connection, except to glean technical information about the product and its functions. The algorithm had been developed and tested in accordance with European laws and regulations, and was marketed as an application with high analytical, clinical and diagnostic accuracy. It was CE-marked (as a Class IIa-product) in the Medical Device Regulation (MDR).

The application was designed to collect patient medical history and triage information in the context of online primary care services via a structured online questionnaire. The questionnaire included both patient-reported structured data and an unstructured free-text field for symptom descriptions. The data were processed using machine learning algorithms and Bayesian networks, probabilistic graphical models that use a directed acyclic graph (DAG) to represent the conditional dependencies and relationships between a set of random variables. The algorithm was adjusted to local data with feedback from engaged professionals in the workplace.

As it was integrated into the booking systems of regional primary healthcare facilities, the AI-based triage application enabled patients to select available appointment slots. The application generated a comprehensive report based on the patient’s input, assessing symptom urgency and suggesting potential differential diagnoses. The AI-generated summary, including the urgency rating, potential diagnoses and the patients’ symptom descriptions, was designed to aid in decision making and referral. The designated healthcare professional contacted the patient to finalize the care plan, and the final decisions rested with the healthcare practitioner. At the time of the study, the AI-based triage application was being implemented and utilized in public primary care facilities across four regions in Sweden, to facilitate care planning and scheduling.

### Participants and recruitment

The data was collected in two primary healthcare settings in southern Sweden. To be eligible for inclusion in the study, participants had to meet the following criteria: healthcare professionals working in primary healthcare within one of the designated regions, have practical experience in implementing and utilizing the AI-based triage application, and demonstrate proficiency in the Swedish language.

The recruitment process was conducted in collaboration with two primary care managers, one in each primary healthcare setting. Initially, managers were consulted to confirm their willingness to allow healthcare professionals with experience of using the application to participate in the study. Purposive sampling was employed to achieve a diversity of study participants. Managers were asked to provide the researchers with contact details of employees of varying gender, occupational background, and level of experience of the application. Subsequently, 15 healthcare professionals of varying genders and professions were contacted and provided with written information (by the second author, ES). One individual declined to participate, resulting in a study population of 14. The study sample included two psychologists, eight physicians, one social worker, and three registered nurses (five men and nine women), most of whom had full-time employment, but only worked part-time with the application. Usage ranged from three weeks to three years, with an average of two years. Five participants had used the AI-based triage application for less than one year, while nine had used it for two to three years.

### Data collection

Semi-structured interviews were conducted via individual video communication between March and May 2023 by two PhD researchers (ES, female and DT, male, a researcher with knowledge of change management), both of whom were trained and experienced in qualitative methods and with an academic interest in implementation within healthcare. Neither researcher had a pre-existing relationship with the interviewees. The interviews followed an interview guide with open-ended questions [43] and aimed to capture healthcare professionals’ experiences of implementing and utilizing the application, including its practice integration and management of integration-related challenges. To pursue further elaboration or clarification of narratives or statements made by the interviewees, the interviewers employed prompts such as, “Could you please elaborate on that?”. To test the interview guide, a pilot interview was performed with a healthcare professional with experience of implementing and utilizing the application, and it did not lead to any modifications. Interviews averaged 41 min in length (ranging from 31 to 55 min, with a total duration of 574 min). All interviews were audio recorded and transcribed verbatim.

### Data analysis

The four NPT constructs and related subconstructs [44] were translated into Swedish by a native speaker, operationalized to fit the data (MN), and discussed (MN, IL) until consensus was reached. In the resulting Swedish “codebook”, NPT constructs made up a priori categories for deductive analysis (Additional File 2, NPT codebook). The analysis was aided by the software NVivo (QSR, version 14). In the first step, MN and IL read all qualitative data iteratively. In the second step, MN and IL independently and separately chose meaning units with a direct bearing on the aim of the study and then condensed them. Condensed meaning units were independently coded and individually sorted into categories based on the four NPT constructs. Iterative discussions between MN and IL led to the preliminary sorting of the condensed meaning units into these categories. In the third step, the remaining authors were invited to contribute with their knowledge and insight; sorting adjustments were made where necessary (aided by the descriptions of related sub-constructs). In the fourth step, the content in each category was condensed to form a preliminary presentation of results by IL and MN and discussed until consensus was reached in the multidisciplinary research group, which comprised two registered nurses (PS, IL), an occupational therapist (MN), a scientist in biomedicine (JN), a sociologist (ES), a pedagogue (LP), and an behavioral economist (PN). After iterative discussions in the wider author group to determine the final text, quotations were used to illustrate each category and translated into English, ensuring their consistency with the original Swedish quotations.

## Results

The findings of this study, framed by NPT, explore healthcare professionals’ experiences of implementing and sustaining an AI-based triage application in primary care, highlighting insights into the challenges faced, strategies employed, and implications for its integration into daily practice.

The first construct of NPT – *Coherence *– emphasizes the sense-making efforts of the healthcare professionals, both individually and collectively, when operationalizing the AI-based triage application. Participants perceived themselves to be knowledgeable about the purpose of the application and the tasks that it would create for them, although some participants thought, in retrospect, that they should have asked more questions when the application was first introduced to them.

*I would just like to understand … what is it (the AI-triage application)? How does it work? I get that it is a model and that it loops back*,* and that it isn’t a decision tree… it seems smart and so on*,* but… (Psychologist #2)*.

The participants’ initial perceptions about the potential benefits and importance of the AI-based triage application for their work were positive: they were interested and excited, for the most part, to try out the new technology and be part of a modern trend. On attempting to distinguish the new way of working from current triage practice, differences were identified.

One such difference was that reading the output of the application replaced listening to patients on the telephone. Also, there were differences related to processing patient information.

*But even if [the AI triage application] has given some suggestions*,* I always look at the guidelines that we used when I worked at the national triage site (Nurse #2)*.

The second construct of NPT – *Cognitive participation* – (the work of building and sustaining engagement) highlights the relational efforts of healthcare professionals in building and maintaining a “community of practice” around the AI-based triage application. The introduction of the AI-based triage application was perceived as uncomplicated, as the technology allowed for a quick grasp of the work involved. The participants perceived digitalization to be an unavoidable trend in both healthcare and society.

The participants considered the implementation of the AI-based triage application to be driven by management. They had differing levels of “buy-in”, even after being involved in developing the AI-based triage application in collaboration with the external tech company that had developed it. Significant time and effort were invested in engaging colleagues in the new practice. However, there were perceptions that the organizational leadership did not fully recognize the extent of work required to establish and sustain the necessary actions and procedures involved in the application. This included the effort to adapt their work to accommodate the new way of working, but also the time invested in helping the company to develop the product. Shortly before the interviews, a decision had been made by senior management to replace the application under investigation with a different one, as part of the organization´s new contract with a vendor of electronic health records. A third party advised them in the choice of which triage application to include in the new solution.

*We have had the present triage application for five years now*,* and now we are going to change the electronic health record for the whole region…One of the companies has taken the initiative to decide which triage system will be part of the new system. Not our organization’s procurers*,* which is completely bizarre! … So*,* the application that we worked to get operational for years will be traded in… (General practitioner #1)*.

Participants described that a solid “community of practice” had not been established, as they perceived the use of the AI-based triage application as optional.

They also questioned their capacity to organize themselves collectively, i.e. to take individual and collective responsibility for the work involved in implementing and using the application. This uncertainty was attributed to a lack of technological know-how in their professional roles, which made it difficult for them to ask the “right” questions.

This uncertainty was attributed to a lack of technological know-how in their professional roles, which made it difficult for them to ask the “right” questions when first presented with the application, and later while using it.

*“Sometimes*,* it is hard to decide what’s what. …We sometimes get a bit “starstruck” by technology… We don’t always express our uncertainty about its format*,* and so on. It was not as if my professional group expressed a desire for more digital patient consultation…There are a lot more questions we would need to have asked” (Psychologist #2)*.

The third construct of NPT – *Collective action* – emphasizes the operational efforts undertaken by healthcare professionals to enact the AI-based triage application. The tasks required by the application included reading the patient-reported description of the problem in free-text, the application-generated report (derived from the patients’ responses to items in the digital questionnaire), an application-generated emergency score, and an application-generated list of potential diagnoses.

The study participants described that the tasks required in the AI-based triage application were only partially performed. Some participants described a lack of trust in the application to allocate patients to the appropriate professional group, and certain aspects of professional roles were not fully reflected in the application, which led to unnecessary consultations. Moreover, the emergency ratings were not perceived as relevant by some professional groups.

Study participants often took a quick glance at the report provided by the application. If they did read the report, they were primarily interested in the patient’s own free-text description of their problem and tended to use this as the basis for their follow-up patient consultations. Having an overview at hand when preparing for a consultation was sometimes experienced as convenient. Participants considered, however, that the report was not fully up to date because a patient’s situation might have changed since time had passed since completing the online questionnaire. They also felt that it was not entirely reliable because some patients had told them that they had difficulty understanding how to respond to questions, or incomplete because some important information was lacking. The participants described that the list of potential diagnoses provided by the AI-based triage application was sometimes perceived as overwhelming, and not always relevant, and therefore of limited practical value as a decision support. They also expressed that the application’s output did not always align with their clinical judgment, experience, and professional training.

*“It [the AI-based triage application] is helpful in some ways*,* but I am still a person who has my own thoughts… I mean*,* I see the suggestions for various diagnoses*,* but although it sometimes makes things easier*,* I don’t rely on it” (General practitioner #3)*.

The participants expressed challenges in the healthcare system’s organizational support for the use of the application, noting that it was not always possible to automatically book patients because there was a lack of available time slots. They pointed out that the absence of established routines for using the application resulted in variations in practice among healthcare professionals. As the application’s output was not directly linked to the patient’s electronic health record (EHR), they chose either to copy all of it into the EHR system, or to only write a summary in their own words, while sometimes they included both.

Participants also expressed concerns about the potential time-lapse in their communication with patients. Because the primary healthcare organization did not offer a 24-hour service, delayed responses were reported to occur. This, in turn, contributed to feelings of discomfort regarding the potential risks associated with delays in patient care.

Additionally, the participants expressed that important guidance regarding how to act on the output of the application was not always available.

*There was an instance where a patient had answered “yes” to the question as to whether they were suicidal*,* were thinking of suicide. …I didn’t know what to do with a patient like that*,* I don’t know the routine. So*,* I called my boss and asked: ok*,* so this patient has answered in this way*,* he or she lives in such and such a place*,* there is an Emergency Unit there*,* but how do we manage this situation?” (Psychologist #1)*.

The fourth construct of NPT – *Reflexive monitoring* – highlights the appraisal efforts that the professionals perceived that they made to assess and understand how the AI-based triage impacted themselves and others.

The study participants reported not being aware of any formal evaluation of the AI-based triage application’s usefulness and effectiveness, but they expressed doubt about it being a time-saver. Participants remarked on the resources invested in introducing and developing the application. They questioned its overall time-effectiveness: even though the AI-generated summary could save time during patient consultation, time still had to be spent reading through the information beforehand. Information was sometimes perceived to be more detailed than in traditional triage situations, although this was mainly the case when patients had been thorough in completing the online questionnaire. Within the standard time for triage (15 min), participants reported that when information was incomplete or missing, they experienced the consultation time as insufficient. In addition, some participants perceived that the application, by design, prioritized patient safety to such a degree that it tended to refer even mild or unclear symptoms to physical consultations. This cautious approach was seen as potentially leading to unnecessary patient consultations. Furthermore, the application was perceived to be designed to cater specifically for medical conditions, even though issues presented by patients in primary care are often of a much more complex character.

*The patient does not really have a medical diagnosis*,* it’s not about that*,* it’s about something else. And*,* of course*,* the patient may present with somatic symptoms*,* but there is no diagnosis. There is such a discrepancy there*,* the application doesn’t manage to grasp that… That’s been my feedback all the time*,* this interminable digging using the application takes a lot of time and the result is far from the target*,* and the patient just gets really tired answering irrelevant questions (General practitioner #3)*.

Some participants raised concerns about the AI-based triage application, questioning its reliability and safety, related to insufficient testing.

*“This is about … choosing to introduce these types of new systems without really testing them and without having enough evidence*,* not enough experience of them*,* and there haven’t been any pilot studies to evaluate them. In research*,* you would never do that*,* you would always need ethical approval and test them in extensive pilot studies… For me*,* as a healthcare professional*,* it just seems incomprehensible that one would just do it in this way – especially in an area in which we have extremely high expectations of evidence in everything we do” (General practitioner #3)*.

Participants reported that they sometimes needed to assist patients in navigating the application, as they observed that some patients struggled to manage it. This meant that some patients were more equipped to navigate into consultation slots than others, while others faced difficulties and were sometimes left without. Additionally, some patients provided less detailed information in their on-line questionnaire, because of language- or other issues.

*“Some write … ‘my child has spots’ or ‘a fever’ and no more. And there can sometimes be language difficulties…so when you access the [the AI-based triage application] it has no information. And if there are other languages involved*,* they very rarely use [the AI-based triage application]*,* they just write “hey*,* hello*,* I want to speak in English”*,* “yes*,* okay”. So*,* in those cases*,* there may be really very complex issues and the history-taking takes maybe 20 minutes or more” (Nurse #3)*.

The online questionnaire was also perceived as rather wearisome for patients. Patients with less digital literacy, a lower level of writing skills, and lower psychological and cognitive capacities were perceived to be disadvantaged. This could be reflected in patients providing more limited information in the patient questionnaire.

*“You know*,* this questionnaire can be very extensive*,* so it’s a burden to patients… It can even scare people away. It’s just a complicated*,* bureaucratic system” (Psychologist #1)*.

The healthcare professionals in this study varied in their appreciation of the AI-based triage application in relation to their own practice. For some, the application did not change their practice, while for others, it was mainly perceived to facilitate the start of patient consultations.

*“I think that it’s good for patients to have the opportunity to start thinking before their visit why they are consulting us …And then*,* when this has already been done*,* we can focus on other aspects of the consultation and then it’s a “win-win” situation for the patient and for us” (General practitioner #2)*.

The participants reported that they not only invested extra time because the AI-based triage application had to be accommodated into their daily practice, but also because they were involved in developing it while using it in their clinical work, through providing feedback about its technical features and functions. Following their recommendations, some features had been added by the external tech company, such as the patient’s own report, and some had been removed, such as making patients fill in the online questionnaire again when registering for a follow-up visit.

## Discussion

This study explores healthcare professionals’ experiences of using an AI-based triage application in primary care, inspired by the framework of normalization process theory (NPT), and focuses on the practice embedment of the application.

The findings suggest that the participants reported diverse and conflicting experiences. While its adoption was largely seen as part of an inevitable shift toward digitalization, professionals experienced a sense of disconnection, highlighting a lack of *Coherence* in its integration. Despite demonstrating *Cognitive Participation* by working to sustain the new practice, the absence of clearly defined collective actions and organizational support created barriers to full engagement. The perception that the AI-based triage application was less reliable than the professionals’ clinical expertise further impeded *Collective Action*, even though substantial time and effort had been invested in its development. *Reflexive monitoring* revealed mixed perspectives – while the application was acknowledged for its role in initiating patient consultations, uncertainty persisted regarding its overall usefulness, effectiveness, and provision of equitable access for all patients.

## Discussion of results

Our qualitative analysis provided an interesting insight into the dynamics of integrating the AI-based triage application into clinical practice through the constructs in NPT. Participants perceived themselves to be knowledgeable about the purpose of the AI-based triage, although also expressed that they should have asked more questions when the application was first introduced to them. At the time, they expressed interest in this new way of working and regarded it as a good idea when considering the operationalization of the application. This demonstrates a sense of *Coherence* in their understanding of the system’s purpose. While initial engagement is an encouraging starting point, research suggests that early enthusiasm alone is not sufficient to guarantee a long-term commitment to change [45, 46]. Despite the critical role of sustainability planning, studies reveal that such planning is often overlooked [47], leading to difficulties in maintaining new practices over time. Moreover, as stakeholders continue to engage with a new method, their perceptions of barriers and facilitators are likely to evolve, underscoring the dynamic nature of implementation processes [48].

Participants reported that, while the AI-based triage application was easy to start using, they faced challenges in defining, planning, and supporting collective actions and procedures necessary to sustain the new practice, and to support a community of practice around it. This difficulty in integrating it into the broader healthcare system highlights issues related to *Cognitive Participation*. The primary healthcare organization aimed to facilitate adoption of the application by using opinion leaders or champions, i.e., respected individuals within the local network who served as role models and involved them as users that assisted the external tech company in refining the AI-based triage application.

While this strategy demonstrated some effectiveness in promoting staff engagement, research suggests that relying solely on opinion leaders is insufficient. Instead, a more systematic approach is needed to ensure the normalization of the practice across the whole cohort of healthcare professionals involved [49, 50].

The findings showed that the AI-based triage application was partially enacted, reflecting challenges in fully integrating it into clinical workflows (i.e., Collective action). Among its various components, the study participants favored the patient-reported description of the problem in free-text over other application components, such as the AI-generated report, emergency score and a list of potential diagnoses. This selective use may reflect that the participants placed greater trust in their own clinical judgment, skills, and training than in the output generated by the AI-based triage application. While they acknowledged the AI-based triage application as effective at a superficial level, they emphasized its limitations in analyzing and categorizing more complex clinical problems. Their reservations may partly be driven by an implicit inclination to retain confidence in the expertise and irreplaceability of their profession, reinforcing a perceived distinction between human clinical reasoning and automated decision making. This tension highlights broader challenges related to meaningful alignment and role coordination in the workplace, which have been shown to be critical to both staff retention [51, 52] and maintenance of quality of care. Professionals’ core responsibilities in contemporary care are likely to continue to be subject to change due to innovations such as AI, but both patients and professionals highlight that human staff’ s capability of adapting care to each individual’ s unique social, existential, cultural and biological circumstances will continue to be a key contribution to quality of care [53, 54]. A sense of professional agency needs to be fostered, to address such concerns, and AI needs to be integrated in a way that enhances, rather than diminishes, clinical expertise. Intrinsic motivation plays a critical role in this process, as it ensures that work tasks are performed “conscientiously, responsibly, and with quality” [55].

The participants in the current study described a lack of awareness regarding any formal evaluations of the effectiveness and safety of the AI-based triage application, which highlights gaps in *reflexive monitoring*. Research shows that, while staff engagement is crucial to successful implementation, significant investments by healthcare professionals without demonstrable success may have substantial long-term adverse effects, which can impact patient care, increased costs, and diminished staff trust in future implementation processes [31, 56]. Implementation research in technology contexts highlights the importance of individuals’ knowledge and beliefs, emphasizing that making the potential benefits transparent through ongoing evaluation and feedback is important [57, 58]. Without structured strategies for continuous feedback of the technology´s effectiveness, healthcare professionals may struggle to see the value of the AI-based triage application, further hindering its normalization within clinical practice.

Study participants expressed how they informally tried to determine the impact of the AI-based triage application on their *professional practice*, on *the organization* and on the *care of patients*.

Some appreciated the potential of the AI-based triage application to facilitate aspects of individual professional practice, while others found it less suited to addressing the broad complexity of medical and health problems in primary care. Nevertheless, there was openness to the potential value of AI in more specialized clinical applications. Research supports this perspective, demonstrating that AI-based clinical decision support systems can enhance diagnostic accuracy and usability in targeted areas, such as melanoma detection [59] and headache diagnosis [60], and thereby foster trust among users.

Beyond the perspectives of healthcare professionals, it is also important to acknowledge that the intentions and business models of technology companies shape how AI-based triage applications are designed, implemented, and sustained in practice. Commercial aspects, such as market viability and return on investment, may not fully align with the clinical priorities of healthcare professionals or with the diverse needs of patients. Such misalignments risk increasing inequalities in access to and use of digital triage, particularly for patients with limited digital literacy or health literacy. Embedding of AI-based triage is therefore not only a matter of professional adaptation but also one of handling the interplay between commercial logics, organizational priorities, and patient capabilities. The outcome of an implementation process is often described as a complex of implementation outcomes, service outcomes and client outcomes [48, 61]. Future research should further examine these dynamics to ensure that implementation strategies address potential risks for unequal outcomes following implementation.

Our participants expressed concerns about the value of the AI-based triage application as a time-saver for the *organization*. Research indicates that the expected time-saving benefits of AI in healthcare are not always evident. The results indicate that participants could use the application effectively only when patients provided sufficient information, but faced challenges when patients struggled to prioritize, or had not prioritized, reporting symptoms. This aligns with Safi’s (2018) study, which found that, while patients appreciated that technology can grant greater autonomy in healthcare decisions, benefits were limited to those who could navigate it effectively [62].

Regarding benefits for *patient care*, our participants raised security concerns that limited the use of the AI-based triage application, including missed important messages linked to staff scheduling and unclear routines for handling unexpected content in the AI-generated reports. While some studies highlight benefits, such as AI-based triage applications with Bayesian reasoning-based techniques for symptom gathering in emergency care [9], others reveal safety risks. Third-party communication, particularly closed-ended questioning, has been linked to severe cases of malpractice [63, 64]. Research on telehealth and teletriage safety remains sparse and inconsistent, highlighting the need for reliable systems to ensure patient safety, as telehealth grows rapidly [65].

In conclusion, the study suggests that the AI-based triage application was not fully integrated into the primary care setting. In our analysis of the interview data, we have explored practice embedment of the application using NPT-constructs. Additional factors, such as workplace culture, job strain, staffing shortages [66, 67] and leadership readiness may also have played a role.

Although policy support for implementation and integration is typically a leadership responsibility, research indicates that leaders often perceive this task as complex. Previous research on AI implementation [23, 68, 69] has identified numerous obstacles, including technical, cultural and contextual challenges for leaders and administrators, along with issues related to evaluation and end-user engagement. Specific barriers for healthcare professionals, identified in a scoping review focused on AI for medical history-taking and triage [20], include negative receptivity to AI tools, a lack of acceptance of AI-generated diagnoses, and insufficient time to learn how to use and adopt new technical systems. These findings align with research demonstrating the multi-level complexity of technology implementation, and the consistency of these challenges across various domains and settings over time [57]. To address these challenges, it is critical to recognize the importance of policy support and the alignment of technology with existing organizational workflows. These factors are essential for the successful implementation and integration of e-health applications in practice [57, 58, 70].

### Strengths and limitations

The trustworthiness of the study can be discussed using the concepts of credibility, dependability, confirmability, and transferability [71].

The credibility of the study was enhanced by involving 14 healthcare professionals with a variety of professional backgrounds, ages, and work experiences. This approach is guided by the concept of “information power”, which posits that a smaller, targeted sample can yield valuable insights, particularly when the study’s objectives are well-defined, high-quality dialogue is prioritized, and the richness of the data influences scientific quality more than its volume [72]. The healthcare professionals’ extensive experience of using the AI-based triage application in primary care facilitated an in-depth exploration of its practice embedment, and provided meaningful insights [71].

Dependability was enhanced by applying a categorization of data based on the NPT. This methodology was valuable because it captured the experiences of using the AI-based triage application in practice. The theoretical constructs of the NPT have been found to have high stability across settings and have shown evidence of functioning as a heuristic device to explain and guide implementation processes [73]. The use of the NPT was complex, however, as the theory proposes that the categories include aspects of normalization at both the individual and organizational (peer-group) levels. A process was undertaken to translate and systematically operationalize the NPT constructs and subconstructs in alignment with the study context, to more accurately connect the interview data into content relating to the various main theory-based categories. It may be important to note that data referring to constructs from the earlier phases of the implementation process (“Coherence” and “Cognitive participation”) may, in theory, be more susceptible to recall bias than those occurring closer to the time of the interview (“Collective action” and “Reflexive monitoring”).

The study is limited in that it focuses on the *mechanisms* of change. May et al. (2022) defines these as “the work that people do when they participate in implementation processes”. In NPT, this work includes coherence building, cognitive participation, collective action, and reflexive monitoring [44]. An account of the influence of the *contexts* in which the implementation work is done, and a more explicit description of how things changed as implementation processes proceeded *(outcomes*), were outside the scope of this study.

The inclusion of researchers with a variety of backgrounds and knowledge was a strategy to ensure confirmability. The research team had both clinical and scientific expertise, including insider knowledge of healthcare practice and extensive experience of qualitative methodology and application of implementation science theory. Confirmability was enhanced by incorporating quotations that reflected the healthcare professionals’ voices, highlighting both the variations and similarities within the data.

As participation required both willingness and time to engage in an interview, the study may have attracted healthcare professionals with particularly strong positive or negative experiences. This limitation is inherent to qualitative interview studies and should be considered when assessing transferability. In addition, the contexts available for sampling were few (because of the relatively few cases in which AI-based triage applications have been utilized over a longer period) and this may restrict the general transferability of the study. Notwithstanding, the interviews yielded a deep and broad scope of data, which, along with the detailed description of the study context, may facilitate transferability to the reader’s context.

## Conclusions

This study found that an AI-based triage application, which appeared to be integrated into primary care practice was only partially embedded into professional and organizational routines. Despite a broad acceptance of digitalization, the healthcare professionals perceived several barriers related to the application, including insufficient organizational and policy support. Additionally, they expressed uncertainty regarding its usefulness, effectiveness, and equitable accessibility for all patients. The findings suggest that further efforts are needed to overcome these barriers and to ensure successful normalization.

## Supplementary Information

Supplementary Material 1.

Supplementary Material 2.

## Data Availability

The datasets used and/or analyzed during the current study are available from the corresponding author on reasonable request.

## Abbreviations

AI: Artificial Intelligence
NPT: Normalization Process Theory
