# Supplementary material for: Integrating AI-based triage in primary care: a qualitative study of Swedish healthcare professionals’ experiences applying normalization process theory
Source: BMC Prim Care. 2025 Nov 4;26:340. doi: 10.1186/s12875-025-03057-9 (PMC12584356; doi:10.1186/s12875-025-03057-9)
Supplement: Supplementary file 1 — Supplementary Material 1. [file 12875_2025_3057_MOESM1_ESM.docx]

Additional File 2.

| **NPT** | **Operationalization in the context of this study*** | **Explanation** |
| --- | --- | --- |
| COHERENCE |  |  |
| Differentiation | Participants distinguish the use of the AI-based triage application from current ways of working. | Whether the AI-based triage application is easy to describe to participants and whether they can appreciate how it differs or is clearly distinct from current ways of working. |
| Communal specification: Sense-making | Participants collectively agree on the purpose of the AI-based triage application. | Whether participants have built or can build a shared understanding of the aims, objectives, and expected outcomes of using the AI-based triage application. |
| Individual specification | Participants individually understand what using the AI-based triage application requires of them. | Whether individual participants have made or can make sense of the work – specific tasks and responsibilities – that the AI-based triage application intervention would create for them. |
| Internalization | Participants construct potential value of using the AI-based triage application for their work. | Whether participants have grasped or can easily grasp the potential value, benefits and importance of using the AI-based triage application. |
| COGNITIVE PARTICIPATION |  |  |
| Initiation | Key individuals drive the use of the AI-based triage application forward. | Whether or not key individuals are able and willing to get others involved in using the AI-based triage application. |
| Enrolment | Participants agree that using the AI-based triage application should be part of their work. | Whether or not participants believe that it is right for them to be involved, and that they can contribute to the implementation work. |
| Legitimation | Participants buy into using the AI-based triage application. | The capacity and willingness of the participants to organize themselves to collectively contribute to the work involved in using the AI-based triage application. |
| Activation | Participants continue to support the use of the AI-based triage application. | The capacity and willingness of participants to collectively define the actions and procedures needed to continue the use of the AI-based triage application. |
| COLLECTIVE ACTION |  |  |
| Interactional Workability | Participants perform the tasks required by the AI-based triage application. | Whether people can enact the AI-based triage application and operationalize its components in practice. |
| Relational Integration | Participants maintain their trust in each other’s work and expertise through the AI-based triage application. | Whether people maintain trust in the AI-based triage application and in each other. |
| Skill set Workability | The work of the AI-based triage application is appropriately allocated to participants. | Whether the work required by the AI-based triage application is seen to be parceled out to participants with the right mix of skills and training to do it. |
| Contextual Integration | The AI-based triage application is adequately supported by its host organization. | Whether the AI-based triage application is supported by management and other stakeholders, policy, money and material resources. |
| 4. REFLEXIVE MONITORING |  |  |
| Systematization | Participants access information about the effects of using the AI-based triage application. | Whether participants can determine the effectiveness and usefulness of the AI-based triage application, from engagement with formal and/or informal evaluation methods. |
| Communal appraisal | Participants collectively assess the AI-based triage application as worthwhile. | Whether, as a result of formal monitoring, participants collectively agree about the worth of the effects of the intervention. |
| Individual appraisal | Participants individually assess the AI-based triage application as worthwhile. | Whether individuals involved with, or affected by, the AI-based triage application think it is worthwhile. |
| Reconfiguration | Participants modify their work in response to their appraisal of the AI-based triage application. | Whether individuals or groups using the AI-based triage application can make changes as a result of individual or communal appraisal. |

* Ingrid LARSSON, Elin SIIRA, Lena PETERSSON, Jens M NYGREN, Petra SVEDBERG, Per NILSEN, Margit NEHER. Integrating AI-based Triage in Primary Care: Swedish Healthcare Professionals’ Experiences through the Lens of Normalization Process Theory. A qualitative study (2025)

**References**

May, C., Rapley, T., Mair, F.S., Treweek, S., Murray, E., Ballini, L., Macfarlane, A. Girling, M. and Finch, T.L. (2015) Normalization Process Theory On-line Users’ Manual, Toolkit and NoMAD instrument. Available from: www.normalizationprocess.org (Accessed May 2024)

May, C.R., Albers, B., Bracher, M. et al. Translational framework for implementation evaluation and research: a normalisation process theory coding manual for qualitative research and instrument development. Implementation Sci 17, 19 (2022).
